# Treatment for Locally Resectable Stage IIIC1 Cervical Cancer: A Retrospective, Single-Institution Study

**DOI:** 10.3390/healthcare11050632

**Published:** 2023-02-21

**Authors:** Yoko Kashima, Kosuke Murakami, Chiho Miyagawa, Hisamitsu Takaya, Yasushi Kotani, Hidekatsu Nakai, Noriomi Matsumura

**Affiliations:** Department of Obstetrics and Gynecology, Faculty of Medicine, Kindai University, Sayama 589-8511, Japan

**Keywords:** cervical cancer, chemotherapy, concurrent chemoradiotherapy, lymph node, surgery, radiotherapy

## Abstract

According to the revision of the FIGO 2018 staging system, cervical cancer with pelvic lymph node metastases was changed to stage IIIC1. We retrospectively analyzed the prognosis and complications of locally resectable (classified as T1/T2 by TNM classification of the Union for International Cancer Control) stage IIIC1 cervical cancer. A total of 43 patients were divided into three groups: surgery with chemotherapy (CT) (ope+CT group) (T1; n = 7, T2; n = 16), surgery followed by concurrent chemoradiotherapy (CCRT), or radiotherapy (RT) (ope+RT group) (T1; n = 5, T2; n = 9), and CCRT or RT alone (RT group) (T1; n = 0, T2; n = 6). In T1 patients, recurrence was observed in three patients, but there was no difference among the treatment groups, and no patients died. In contrast, in T2 patients, recurrence and death were observed in nine patients (8 in ope+CT; 1 in ope+RT), and recurrence-free survival and overall survival were lower in the ope+CT group (*p* = 0.02 and 0.04, respectively). Lymphedema and dysuria were more common in the ope+RT group. A randomized controlled trial comparing CT and CCRT as an adjuvant therapy after surgery in T1/T2 patients, including those with pelvic lymph node metastases, is currently underway. However, our data suggest that performing CT alone after surgery in T2N1 patients is likely to worsen the prognosis.

## 1. Introduction

Cervical cancer is the fourth most common cancer among women, with 604,000 reported patients and 342,000 deaths worldwide in 2020 [1]. In Japan, the number of patients with cervical cancer is increasing among young women in their 50s or younger [2]. Although cervical cancer cases are expected to decrease worldwide with the increased human papillomavirus (HPV) vaccination [3,4], HPV vaccination rates are low in Japan [5].

In cervical cancer, lymph node (LN) metastasis is an important prognostic factor [6,7,8,9]. According to the latest International Federation of Gynecological Organizations classification (FIGO 2018), if the tumor reaches the lower third of the vagina, extends to the pelvic wall, or if there is lymph node metastasis and no distant metastasis, it is classified as stage III. In addition, if there is metastasis in the pelvic lymph nodes by imaging or pathology, it is subclassified as stage IIIC1. Patients with para-aortic LN metastasis (N2) are now classified as stage IIIC2 [10]. Concurrent chemoradiotherapy (CCRT) has substantially improved both the recurrence-free survival (RFS) and overall survival (OS) of locally advanced cervical cancer with LN metastases and has become the international standard of care. For example, the National Comprehensive Cancer Network 2022 guideline recommends external pelvic beam radiation therapy and brachytherapy with platinum regimens as CCRT for patients who are positive for pelvic LN metastases on a surgical biopsy [11]. Furthermore, if the results of imaging studies are positive for pelvic LN metastases and are negative for para-aortic LN metastases, a surgical biopsy of the para-aortic LN is recommended, followed by an extended irradiation if para-aortic LN metastases are pathologically confirmed [11]. Radiotherapy (RT)-based treatment is less invasive than surgery and can be safely performed in elderly patients and those with multiple comorbidities. RT can avoid the urinary dysfunction that is associated with extensive surgical resection.

In the Japanese guidelines in 2011 and 2017, a radical hysterectomy was recommended for patients corresponding to stage IB-II of the FIGO 2008 classification (FIGO 2008), regardless of the presence or absence of LN metastases [12,13]. Additionally, neoadjuvant chemotherapy (NAC) was listed as an option [12,13]. Therefore, in Japan, surgery has been the main treatment for patients whose tumor is locally resectable (classified as T1/T2 by TNM classification of the Union for International Cancer Control), even for the FIGO 2018 stage IIIC. In addition, chemotherapy (pre-operative and post-operative), RT, or CCRT have been used as adjuvant therapies. In Japan, the staging was revised in 2020 following the FIGO 2018, and the new treatment guidelines were issued in 2022. However, no clear recommendation has been made as to whether surgery or CCRT should be selected as the main treatment for patients with stage IIIC [14]. Surgery has the following advantages: the accurate detection of disease extent based on pathological diagnosis, the treatment of tumors refractory to chemotherapy and radiotherapy, and the preservation of ovarian function in young patients.

Until early 2018, in our institution, an NAC plus radical hysterectomy was generally performed for patients with tumor diameters greater than 4 cm. However, there are reports that the NAC plus radical hysterectomy has a significantly lower disease-free survival rate than CCRT [15,16], and NAC has not been performed since then. As for adjuvant therapy, CCRT has generally been performed in patients who are at a very high risk of recurrence with chemotherapy alone, such as those with a positive or questionable surgical margin, while chemotherapy alone has been used in other cases. Although radical surgery is not recommended internationally for stage IIIC patients [11], there is currently no clear evidence for the treatment of patients with locally resectable T1/T2 tumors with pelvic LN metastases. In this study, we retrospectively examined the prognosis and complications of each treatment for stage IIIC1 cervical cancer, especially in patients with T1 or T2. To the best of our knowledge, this is the first report comparing surgery without RT and RT-based treatment for FIGO 2018 stage IIIC1 cervical cancer.

## 2. Materials and Methods

Among cervical cancers initially treated at Kindai University Hospital between January 2013 and March 2021, we included those with FIGO 2008 stage IA2 to stage IIB. Neuroendocrine carcinomas were excluded. Among eligible patients with FIGO 2018 stage IIIC1. Those with Union for International Cancer Control (UICC) T1/T2 were selected [17]. Age, histopathology, first-line treatment, RFS, OS, and treatment-related complications were retrospectively evaluated.

Patients were divided into three groups according to treatment: the ope+CT group (surgery and chemotherapy (neoadjuvant and/or adjuvant)), the ope+RT group (surgery followed by CCRT or RT), and the RT group (CCRT or RT without surgery). In addition, patients were divided into four groups according to whether they had squamous cell carcinoma (SCC) or non-squamous cell carcinoma (non-SCC) and treatment with or without RT (CCRT or RT). Complications caused by treatment, leg lymphedema, and dysuria at least one month after completion of the initial treatment were investigated. Leg lymphedema was defined based on their medical records. Dysuria was defined as the administration of medication or self-catheterization.

Statistical analyses were performed using GraphPad Prism version 9.4.1 (GraphPad Software, San Diego, CA, USA), the Kruskal–Wallis test for the age distribution. Survival curves were estimated by the Kaplan–Meier method and compared by the log-rank test. Fisher’s exact test was performed using R version 4.2.2 for complication frequency. *p* values less than 0.05 were considered statistically significant.

This study was approved by the Institutional Review Board of Kindai University Faculty of Medicine (R04-186). Patients in this study were allowed to refuse to participate in the survey by opting out on the website of the Kindai University Faculty of Medicine (https://www.kindai.ac.jp/medicine/ (accessed on 18 February 2023)).

## 3. Results

A total of 213 patients were FIGO 2008 stage IA2-IIB, with a median age of 55 (25–97) years; the histological subtype was SCC in 145 patients (68%), adenocarcinoma in 55 patients (26%), and adenosquamous cell carcinoma in 13 patients (6%). Of the 213 patients, 61 (28%) were FIGO 2018 stage IIIC1, including 12 patients with T1 (Table 1) and 31 patients with T2 (Table 2). Among patients with T1 and T2 cancer, 33 (77%) had SCC and 10 (23%) had non-SCC, with a median age of 47 (25–73) years and a median follow-up of 52 (10–111) months (Table 1 and Table 2).

**Figure 1:**
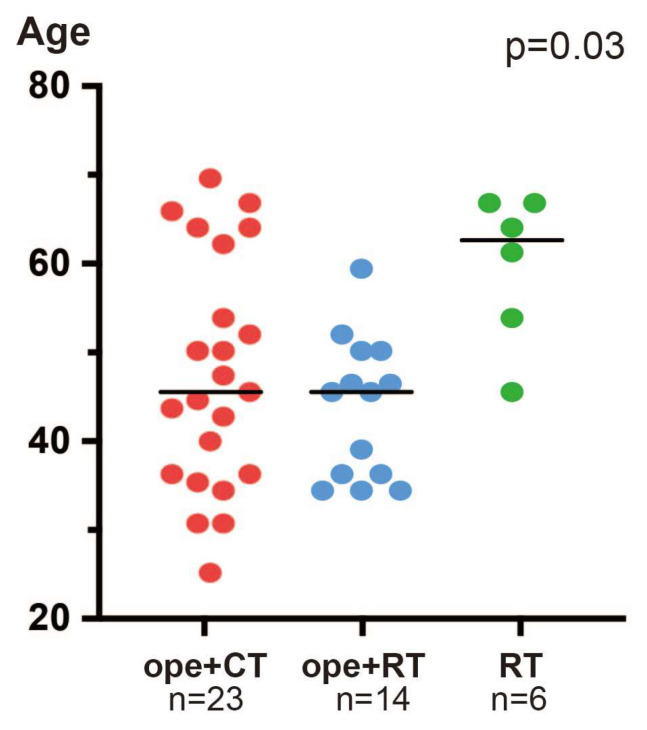
Patient ages by treatment group. ope+CT, patients who underwent surgery and chemotherapy (neoadjuvant or adjuvant); ope+RT, patients who underwent surgery followed by radiotherapy (including concurrent chemoradiotherapy); and RT, patients who underwent radiotherapy (including concurrent chemoradiotherapy). The *p*-value is based on the Kruskal–Wallis test.

The RT group had an older mean age than the ope+CT or ope+RT groups (*p* = 0.02; Figure 1). After October 2018, when the treatment strategy changed, only two of the six patients in the RT group were older than 70 years.

**Figure 2:**
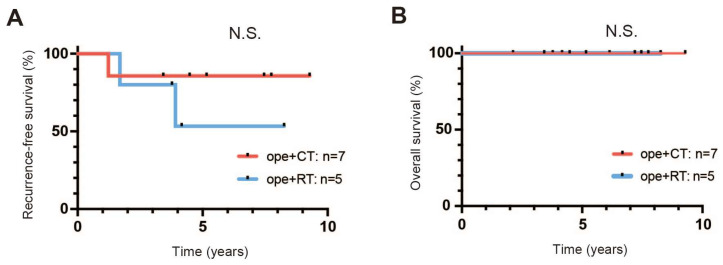
Prognosis of patients with (tumor and node) T1N1. (**A**) Recurrence-free survival (RFS). (**B**) Overall survival (OS). ope+CT, patients who underwent surgery and chemotherapy (neoadjuvant or adjuvant); ope+RT, patients who underwent surgery followed by radiotherapy (including concurrent chemoradiotherapy). N.S.: not significant. Survival curves were estimated by the Kaplan–Meier method and compared using the log-rank test.

Among the stage IIIC1 patients, all those with T1N1 underwent surgery as the main treatment: five in the ope+RT group (surgery followed by CCRT in four patients and surgery followed by CCRT and systemic chemotherapy in one patient) and seven in the ope+CT group (surgery followed by adjuvant chemotherapy in six patients and NAC followed by surgery and adjuvant chemotherapy in one patient (case12)) (Table 1). Recurrence occurred in three patients, with metastases to the vagina, lung, and mediastinal LN, all of which responded to treatment of the recurrent tumor. There was no significant difference in RFS and OS between the ope+CT group and the ope+RT group (Figure 2).

**Figure 3:**
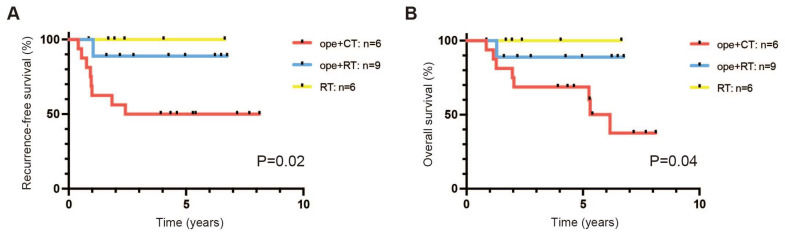

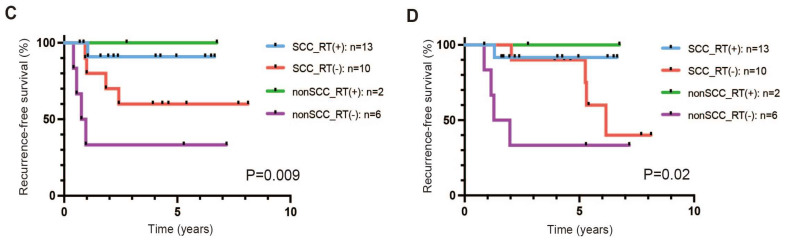
Prognosis of patients with (tumor and node) T2N1. (**A**) Recurrence free survival (RFS). (**B**) Overall survival (OS). ope+CT, patients who underwent surgery and chemotherapy (neoadjuvant or adjuvant); ope+RT, patients who underwent surgery followed by radiotherapy (including concurrent chemoradiotherapy); and RT, patients who underwent radiotherapy (including concurrent chemoradiotherapy). (**C**) RFS by histological subtype. (**D**) OS by histological subtype. Patients were divided according to whether they had squamous cell carcinoma (SCC) or non–squamous cell carcinoma (non–SCC), and whether radiotherapy (including concurrent chemoradiotherapy) was performed or not. Survival curves were estimated by the Kaplan–Meier method and compared by the log–rank test.

Among stage IIIC1 patients with T2N1, nine were in the ope+RT group (surgery followed by CCRT in three patients, surgery followed by RT alone in one patient, NAC followed by surgery and RT alone in one patient (case 23), and NAC followed by surgery and CCRT in four patients (case 24–27)) (Table 2). A total of six patients were in the ope+CT group (surgery followed by adjuvant chemotherapy in five patients and NAC followed by surgery and adjuvant chemotherapy in eleven patients (case 33–43)), and six patients were in the RT group (all received CCRT) (Table 2). Recurrence was observed in eight patients in the ope+CT group and one patient in the ope+RT group; all nine of these patients had a relapse site in the pelvis and died of the disease. There were significant differences in the RFS and OS among the three groups (*p* = 0.02; *p* = 0.04, respectively; Figure 3A,B). Similarly, patients who did not receive CCRT or RT alone had a worse prognosis in terms of both RFS and OS among SCC and non-SCC groups (*p* = 0.009, 0.02; Figure 3C,D).

**Figure 4:**
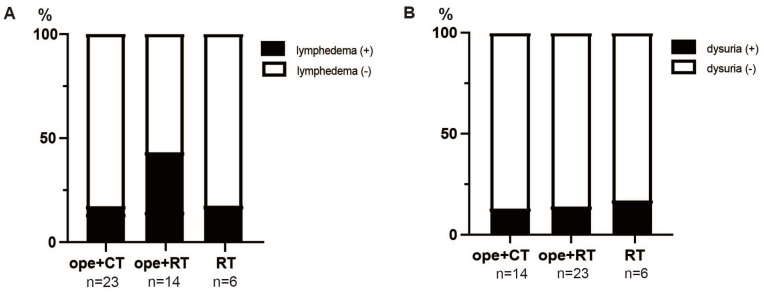
Post–treatment complications in patients with (tumor and node) T1/T2N1. (**A**) Number of leg lymphedema occurrences. (**B**) Number of dysuria occurrences. ope+CT, patients who underwent surgery and chemotherapy (neoadjuvant or adjuvant); ope+RT, patients who underwent surgery followed by radiotherapy (including concurrent chemoradiotherapy); and RT, patients who underwent radiotherapy (including concurrent chemoradiotherapy). Complication frequency was performed the Fisher’s exact test.

As for complications, in patients with stage IIIC1 T1/T2, lymphedema occurred in 17% (4/23) of the patients in the ope+CT group, 43% (6/14) in the ope+RT group, and 17% (1/6) in the RT group; dysuria occurred in 13% (3/23) of patients in the ope+CT group, 14% (2/14) in the ope+RT group, and 17% (1/6) in the RT group. Lymphedema was more common in the ope+RT group, although there were no significant differences between the groups for both lymphedema and dysuria (Figure 4).

## 4. Discussion

The policy at our institution has been to perform surgery whenever possible for patients with resectable T1 or T2 cervical cancer, even if they are FIGO 2018 stage IIIC1. Thus, the older age in the RT group reflects the fact that RT was performed instead of surgery in older patients with poor surgical tolerance (Figure 1). However, since late 2018, an increasing number of patients with T2 have been treated with CCRT as the primary therapy, regardless of their surgical tolerance.

In our cohort, there were no recurrences or deaths and few complications in patients who received CCRT but not surgery (Figure 3 and Figure 4). In 2005, 23 patients with T1b-2a, who were found to have LN metastases intraoperatively and did not undergo hysterectomy, were reported to have a significantly lower 2-year disease-free survival rate than 35 patients who underwent a hysterectomy and had confirmed post-operative LN metastasis [18]. However, a more extensive cohort analysis in 2021 found no significant difference in recurrence or mortality between 361 patients with T1a-2b cancer who underwent a planned hysterectomy and 154 who abandoned the procedure due to intraoperative detection of LN metastases [19]. The analysis showed no survival benefit from a hysterectomy in any subgroup [19]. In addition, a single-center retrospective study in Japan reported that 24 patients with T1b-2b SCC and LN metastases who received CCRT as primary therapy without surgery had RFS and OS that were comparable to those of 45 patients who underwent surgery [20]. These results suggest that a hysterectomy may not be necessary for patients with T1/T2 cervical cancer and pelvic LN metastasis.

Even if a radical hysterectomy is performed for cervical cancer, a recent phase III clinical trial rejected the strategy of NAC for locally advanced patients [15]. In our study, all patients who died of the disease had T2 cancer, including five who underwent NAC followed by surgery and adjuvant chemotherapy and one with NAC followed by CCRT (Table 2). Unless there is new evidence in the future to prove the usefulness of NAC, NAC is not likely to be a preferred option.

When pelvic LN metastases are detected after surgery, CCRT has a better prognosis than RT as adjuvant therapy [21] and is the standard of care. However, surgery followed by CCRT is associated with a lower quality of life, with more leg lymphedema, dysuria, rectal dysfunction, sexual dysfunction, and mental impairment than surgery alone [22,23]. In our study, leg lymphedema also tended to be more common in the ope+RT group, although there were no significant differences due to the small number of patients (Figure 4). In the phase II JGOG1067 trial, 62 patients with cervical cancer and LN metastasis received adjuvant chemotherapy with irinotecan and nedaplatin. The result was favorable, with a 5-year RFS of 77% and lymphedema in only 10% of patients [24]. The phase III JGOG1082 trial (AFTER trial) is currently ongoing and is comparing CCRT and chemotherapy (paclitaxel plus carboplatin or cisplatin) as adjuvant therapies in FIGO 2018 stage IB-IIB patients with pelvic LN metastases and/or parametrial invasion [25].

Our study showed no death in patients with T1N1 cancer, regardless of the treatment. However, the ope+CT group had a worse prognosis in patients with T2N1 (Figure 3). Knowing that post-operative CCRT increases the risk of complications [22,23], we selected CCRT in patients with a strong tendency toward invasion and a predicted high risk of recurrence. Therefore, the selection bias would be present in that surgery followed by CCRT, which would have a worse prognosis. Surprisingly, however, patients with T2N1 in the ope+CT group (with adjuvant chemotherapy) had a worse prognosis than those in the ope+RT and even the RT group, regardless of the histological subtype (Figure 3C,D). Although non-SCC is less radiosensitive than SCC [26,27], the efficacy of CCRT for patients with LN metastases has been demonstrated, even for non-SCC [28,29].

CCRT without surgery is the standard treatment for T2 [11], and therefore, the evidence for surgery followed by chemotherapy for T2N1 cancer is extremely limited in clinical trials and real-world data. In addition, a limitation of this study is that we used retrospective data for a limited number of patients at a single institution. However, the study data for patients with T2N1 cancer strongly suggest the risk of not using RT for T2N1. The JGOG1082 trial aims to test the efficacy of post-operative systemic chemotherapy, including in T2N1 [25]. This trial may clarify the results of our study.

In conclusion, CCRT without surgery may not worsen the prognosis for patients with FIGO 2018 T1/ T2 stage IIIC1 cervical cancer. Surgery with chemotherapy instead of CCRT as the main therapy or adjuvant therapy in patients with T2N1 cancer may worsen their prognosis. In the future, the JGOG1082 trial or other cohort studies may reveal the risks of not using RT for T2N1.

## Figures and Tables

**Table 1:** Stage IIIC1 cases classified as T1 by the TNM classification of the Union for International Cancer Control.

Case	Age	Histological Type	Treatment	Recurrence	Time to Recurrence (Month)	Treatment at Recurrence	Prognosis	Lymph Edema	Dysuria
1	37	SCC	ope+RT	-			NED	+	
2	35	SCC	ope+RT	-			NED	+	
3	54	SCC	ope+RT	+	20	ope	NED		
4	40	AdS	ope+RT	+	46	ope	NED		
5	35	AdS	ope+RT	-			NED	+	+
6	25	SCC	ope+CT	-			NED		
7	31	SCC	ope+CT	-			NED		
8	31	SCC	ope+CT	-			NED		
9	44	SCC	ope+CT	-			NED		
10	47	SCC	ope+CT	-			NED		
11	49	SCC	ope+CT	+	14	RT	NED	+	
12	41	SCC	ope+CT	-			NED		

SCC: squamous cell carcinoma, AdS: adenosquamous cell carcinoma, ope: operation, RT: radiotherapy, CT: chemotherapy, NAC: neoadjuvant chemotherapy, NED: no evidence of disease, OS: overall survival, and RFS: recurrence-free survival.

**Table 2:** Stage IIIC1 cases classified as T2 by the TNM classification of the Union for International Cancer Control.

Case	Age	Histological Type	Treatment	Recurrence	Time to Recurrence (Month)	Treatment at Recurrence	Prognosis	Lymph Edema	Dysuria
13	56	SCC	RT	-			NED		
14	47	SCC	RT	-			NED		
15	64	SCC	RT	-			NED		
16	67	SCC	RT	-			NED	+	+
17	70	SCC	RT	-			NED		
18	70	SCC	RT	-			NED		
19	47	SCC	ope+RT	-			NED		
20	52	SCC	ope+RT	-			NED		
21	48	Ad-G	ope+RT	-			NED		
22	47	Ad-E	ope+RT	-			NED	+	
23	52	SCC	ope+RT	-			NED		
24	35	SCC	ope+RT	-			NED	+	+
25	48	SCC	ope+RT	-			NED	+	
26	62	SCC	ope+RT	-			NED		
27	37	SCC	ope+RT	+	12	BSC	DOD		
28	65	SCC	ope+CT	-			NED		
29	37	SCC	ope+CT	+	28	ope	DOD		
30	46	Ad	ope+CT	-			NED		
31	52	Ad	ope+CT	+	4	RT	DOD		
32	56	Ad	ope+CT	+	9	RT	DOD	+	
33	35	SCC	ope+CT	-			NED		
34	67	SCC	ope+CT	-			NED		
35	52	SCC	ope+CT	-			NED		+
36	54	SCC	ope+CT	-			NED		+
37	70	SCC	ope+CT	-			NED		
38	36	SCC	ope+CT	+	11	RT	DOD		
39	45	SCC	ope+CT	+	22	RT	DOD	+	
40	67	SCC	ope+CT	+	11	RT	DOD		
41	69	Ad-C	ope+CT	-			NED		
42	37	Ad	ope+CT	+	6	RT	DOD		
43	73	Ad	ope+CT	+	11	BSC	DOD	+	+

SCC: squamous cell carcinoma, AdS: adenosquamous cell carcinoma, Ad-G: gastric adenocarcinoma, Ad-E: endometrioid adenocarcinoma, Ad: usual-type endocervical adenocarcinoma, Ad-C: clear cell adenocarcinoma, DOD: died of disease, ope: operation, RT: radiotherapy, CT: chemotherapy, NAC: neoadjuvant chemotherapy, NED: no evidence of disease, OS: overall survival, RFS: recurrence-free survival, and BSC: best supportive care.

## Data Availability

The data that support the findings of this study are available from the corresponding author.
